# Bioactive Natural Antivirals: An Updated Review of the Available Plants and Isolated Molecules

**DOI:** 10.3390/molecules25214878

**Published:** 2020-10-22

**Authors:** Syam Mohan, Manal Mohamed Elhassan Taha, Hafiz A. Makeen, Hassan A. Alhazmi, Mohammed Al Bratty, Shahnaz Sultana, Waquar Ahsan, Asim Najmi, Asaad Khalid

**Affiliations:** 1 Substance Abuse and Toxicology Research Centre, Jazan University, Jazan 45142, Saudi Arabia; mtaha@jazanu.edu.sa (M.M.E.T.); haalhazmi@jazanu.edu.sa (H.A.A.); akahmed@jazanu.edu.sa (A.K.); 2 Department of Clinical Pharmacy, College of Pharmacy, Jazan University, Jazan 45142, Saudi Arabia; hafiz@jazanu.edu.sa; 3 Department of Pharmaceutical Chemistry, College of Pharmacy, Jazan University, Jazan 45142, Saudi Arabia; malbratty@jazanu.edu.sa (M.A.B.); wmohammad@jazanu.edu.sa (W.A.); anajmi@jazanu.edu.sa (A.N.); 4 Department of Pharmacognosy, College of Pharmacy, Jazan University, Jazan 45142, Saudi Arabia; shmali@jazanu.edu.sa

**Keywords:** HIV, HSV, Hepatitis C virus, influenza virus, COVID-19, plant extracts, phytochemicals

## Abstract

Viral infections and associated diseases are responsible for a substantial number of mortality and public health problems around the world. Each year, infectious diseases kill 3.5 million people worldwide. The current pandemic caused by COVID-19 has become the greatest health hazard to people in their lifetime. There are many antiviral drugs and vaccines available against viruses, but they have many disadvantages, too. There are numerous side effects for conventional drugs, and active mutation also creates drug resistance against various viruses. This has led scientists to search herbs as a source for the discovery of more efficient new antivirals. According to the World Health Organization (WHO), 65% of the world population is in the practice of using plants and herbs as part of treatment modality. Additionally, plants have an advantage in drug discovery based on their long-term use by humans, and a reduced toxicity and abundance of bioactive compounds can be expected as a result. In this review, we have highlighted the important viruses, their drug targets, and their replication cycle. We provide in-depth and insightful information about the most favorable plant extracts and their derived phytochemicals against viral targets. Our major conclusion is that plant extracts and their isolated pure compounds are essential sources for the current viral infections and useful for future challenges.

## 1. Introduction 

A virus is a tiny parasite that has no capacity to replicate itself. Once infected in a host agent or living cell, it produces more viruses using host machinery. With their complexity and diversity, it survives for a long time in the host, bypassing the treatments, and it causes devastating issues such as pandemics [1]. They have RNA or DNA as genetic material with single or double-stranded nucleic acid. Using unique physical properties such as phospholipid layers, ligands, and configurations, they invaded into host cells easily [2]. Viral infections can lead to acute as well as chronic conditions. Acute infections happen in an out of balance way; for instance, it is a non-equilibrium process whereby the virus and host change its process until the destruction of the host or control over the infection. The ineffective function of specific genes related to the immunity of the host or effective reduction of host immunity by the viral genes is a niche in this infection and leads to the development of overwhelming consequences [3]. On the other hand, chronic viral infections occur in metastable equilibrium with viral and host genes balancing one another. Sometimes, the virus can persist in the healthy and immune host, which is deprived of any sign of infection [4].

Viral infections and associated diseases are responsible for a substantial number of mortality and public health problems around the world. Each year, infectious diseases kill 3.5 million people worldwide [5]. Even though there are different therapeutic strategies available in the clinical practice, a lack of specificity toward the virus, and the limited efficacy of drugs makes the vaccines a gold standard prophylactic to viral infections. Moreover, the synthetic drugs often do not meet the treatment expectation via either unwanted drug side effects or drug resistance to nucleoside analogues via mutation [6,7]. The drug failure and resistances have led to a growing interest in natural products, especially plants, and investigation into antiviral agent discovery. According to the World Health Organization (WHO), 65% of the world population is in the practice of using plants and herbs as part of the treatment modality [8]. Human use of plants as medicine, including viral infections, dates back 60,000 years to the Paleolithic age [9]. Hence, plants have an advantage in drug discovery based on their long-term use by humans, and lesser toxicity and plenty of bioactive compounds can be expected from them.

Hence, the aim of the present retrospective review is an update on the discovery regarding different plants and lead compounds isolated from them against the essential and clinically significant virus such as the human immunodeficiency virus (HIV), herpes simplex virus (HSV), influenza, and hepatitis c, clarifying their indication with viruses and mechanisms of action. 

## 2. Methodology

To obtain the appropriate literature, we have used relevant keywords such as plants, viruses, phytochemicals, HIV, HSV, influenza, hepatitis-c, HIV integrase, HIV-reverse transcription, HIV-protease, virucidal action, virus replication imbibition, viral attachment, inhibition of hemagglutination, HCV infection replication, etc. These keywords were searched in relevant databases such as Google Scholar, PubMed, Scopus, Scielo, etc. We have collected information from research articles, review articles, PhD theses, books, chapters, and conference abstracts from 1975 to 2020. A total of 207 species have been reported in this review article. The taxonomy of the plant species was properly identified from http://www.theplantlist.org and http://www.ipni.org websites. 

## 3. Human Immunodeficiency Virus (HIV) 

The main target of the human immunodeficiency virus (HIV) is our immune system, where it affects and destroys the immune system function. At present, more than 35 million people are suffering from HIV; so far, it caused more than 39 million HIV-related deaths worldwide [10]. The overwhelming adverse effects of HIV continue globally. The treatment for HIV involves antiretroviral therapy (ART), which is a combination of HIV medicines. Since the year 2000, there has been a significant decrease in HIV-related patient deaths, which accounted for about 50% of all cases. This achievement maybe because of the successful use of ART regimen among the patients and new preventive strategies [11]. Regardless of this progress in HIV treatment with ART and the global measures taken for HIV prevention still, the newly infected HIV patient numbers have been increasing with the rate of 2 million every year [12].

**Figure 1:**
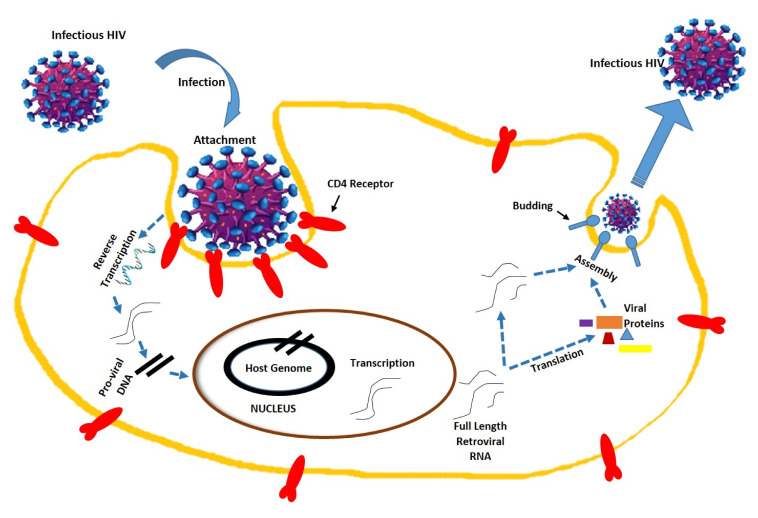
Human immunodeficiency virus structure and replication mechanism. The HIV structure in this figure has been modified from the source www.istockphoto.com.

HIV is a member of the genus Lentivirus, part of the family Retroviridae [13]. HIV contains two copies of single-strand RNA, which is the contributory agent of acquired immunodeficiency syndrome (AIDS) by a progressive decline of the immune system. In this condition, the infections take advantage of the weaker immune system, where the immune system is no longer in a stage to fight back. HIV is an enveloped positive-sense virus, which is meticulously focused on the immune system by infecting CD4^+^ T cells [14,15]. This T helper cell is the core of the immune system, whereby it handles signal transduction toward the rest of the immune cells and thereby protects the whole system against life-threatening infections and endangering subjects. The first stage of infection is the attachment of HIV to the CD4^+^ lymphoid cell surface. After the viral capsid enters the cell, reverse transcriptase liberates a positive sense single-stranded RNA, coping it into a complementary DNA. Then, the nuclei of host cells become integrated with the viral RNA. The integrated DNA is then transcribed into RNA in the presence of transcription factors such as NF-kB, which is then spliced into messenger RNA (mRNA) [16,17,18]. Then, the structural protein is generated and made into a new virus particle (Figure 1).

The antiviral treatments explicitly target these key areas of virus multiplication. Nonetheless, the infection rate of HIV is increasing in spite of ART [19]. Moreover, the ART has become more important, since there is no vaccine available against HIV. However, again, ART is not a panacea for HIV, due to the various side effects and resistance [20,21]. Hence, significant attempts have been employed by natural product biologists to find an alternative for ART. Even the WHO suggests and supports these initiatives. Many plants and plant products such as secondary metabolites have shown significant effects in these targets [22].

Natural products have been explored in finding anti-HIV agents with a critical focus in four mechanisms. They are HIV integrase strand transfer inhibitors [23], Nucleoside Reverse Transcriptase Inhibitors (NRTIs), Nonnucleoside Reverse Transcriptase Inhibitors (NNRTIs), and Protease Inhibitors (PIs) [24]. Integrase is a key enzyme by which HIV inserts (integrate) its viral DNA (proviral) into the DNA of the host CD4 cell. Thus, inhibiting the integrase in the cellular level is a significant target for anti-HIV drug discovery [25]. As per the Food and Drug Administration (FDA), Raltegravir was the first integrase strand inhibitor (INSTI) to be approved in 2007, followed by elvitegravir in 2012 and dolutegravir in 2014. [26]. Natural product discovery has been conducted much time by specifically inhibiting the integrase target [27]. Another target of anti-HIV drugs is reverse transcriptase inhibitors. The reverse transcriptase, a RNA-dependent DNA-polymerase, has been used by the virus to convert RNA to DNA, which is called reverse transcription. Hence, blocking reverse transcription will inhibit HIV replication [28]. In the last phase of viral replication, a viral protease is necessary for the cleavage of a large precursor polyprotein. This cleavage of a protein precursor is crucial for the viral particle maturation and infectivity. Saquinavir, indinavir, ritonavir, and nelfinavir are a few examples of approved protease inhibitors by the WHO [29,30]. Thus, inhibiting protease is also considered as a significant target of anti-HIV natural products.

In our search for natural products in the mentioned databases, we have observed that the majority of the natural products are evaluated for anti-HIV properties up to the crude extraction level only. So, we found a few major secondary metabolites isolated from plants, which have good activity against HIV. A list of plant species with inhibition studies is summarized in Table 1.

The screening of medicinal plants has delivered plenty of secondary metabolites with anti-HIV properties. They include alkaloids, triterpenoids, flavonoids, coumarins, phenolics, tannins, saponins, phospholipids, xanthones, quinones, etc. [81]. There is a large pool of natural compounds with diverse structures, which target different viral targets. Some of them have been found to inhibit HIV integrase and some show RT inhibition (Table 2). The compounds for which we could not establish the mechanism of action will not be included in this review.

## 4. Herpes Simplex Virus

The herpes simplex virus (HSV) infection, otherwise known as genital herpes (GH), is the most frequent cause of genital ulceration worldwide. In general, herpes can appear commonly in the mouth and genitals. The primary cause of oral herpes is the HSV-1 type strain, but genital herpes is commonly caused by the HSV-2 type strain [101]. HSV-seronegative persons (vulnerable group) develop a primary infection on their first HSV-1 or HSV-2 exposure. HSV-1 and HSV-2 are normally spread by different routes and affect different areas of the body, however, the signs and symptoms that they cause overlap. The infection happens through primary contact with mucocutaneous surfaces of an infected person, whereas the virus enters the nerve cells to create latency in the sacral dorsal root ganglion and lesions at the point of entry. Even though HSV is rarely fatal, most people who have been infected and dormant viruses can reactivate; thus, an extensive of HSV pool is available to spread to vulnerable individuals in the society [102]. The estimated worldwide prevalence of HSV-1 is 67%, whereas HSV-2 is less common, infecting ~11% of the world population with the highest prevalence in Africa [103].

**Figure 2:**
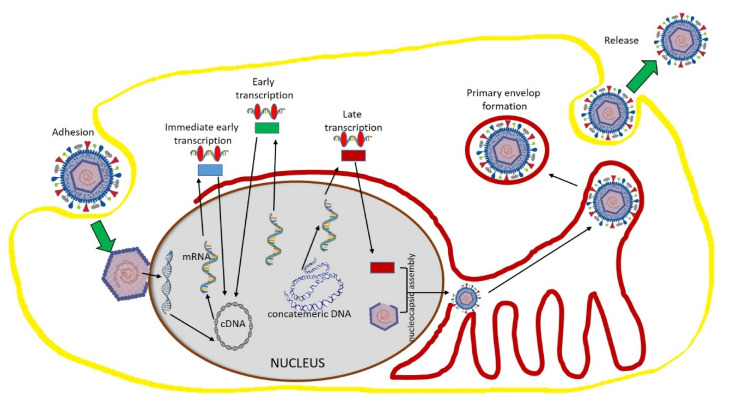
Herpes simplex virus structure and replication mechanism. The HSV structure in this figure has been modified from the source https://pnghut.com.

HSV is a member of Herpesviridae, which is a large family of enveloped double-stranded DNA viruses that causes diseases in both human and animals [104]. Even though Herpesviridae viruses vary in tissue tropism and host interaction mechanisms, they have a much-conserved tool by which they replicate their DNA in infection. Among the members of this family, HSV has been much exploited to study its mechanism of replication. It is well understood that other viruses of this family follow similar replication pathways, but they differ in the pace of activity [105]. Initially, the host cell attachment happens with the HSV virus. This attachment occurs at the heparan sulfate moieties of cellular proteoglycans with the glycoprotein present in the virus envelope, where they bind with the secondary cellular receptors. After the attachment, the viral envelope is released into the cytosol. This will facilitate the movement of capsid toward the nuclear pore, where the viral DNA will be released via the capsid portal. Once in the nucleus, viral DNA transcription leads to mRNA by cellular RNA polymerase II. This viral gene expression is tightly regulated, which is comprised of three kinetic expressions such as early, intermittent, and late mRNA formation. All mRNA transcripts are translated into proteins and travel into the nucleus from the cytoplasm. Capsid proteins assemble in the nucleus to form empty capsids. Then, the newly formed capsids are released from the nucleus to the cytoplasm, where they form its final vesicles [106,107]. Then, the formed virus accumulates in the endoplasmic reticulum and is subsequently released by exocytosis (Figure 2).

There is no ultimate cure for HSV, but the current strategies are mainly focused on symptomatic relief. Both innate and adaptive immune systems can control HSV infections. In fact, the nature of HSV infection is dependent upon how the virus bypasses the host innate immune system. In the current system of practice, antiviral drugs are classified as virucidal, immunomodulators, and chemotherapeutic agents [108]. There is a starting treatment for HSV with acyclovir, valacyclovir, or famciclovir for 7–10 days for primary HSV infections [109]. After that, the treatment will be started only when the recurrence of HSV occurs, and the treatment will be episodic for five days to prevent the symptoms and prevent recurrence [110]. These drugs act via a mechanism of inhibition of DNA polymerase. Even though these drugs are in practice, they can fail to meet the treatment expectation via either unwanted drug side effects or drug resistance to nucleoside analogues via mutation. Therefore, clinicians and microbiologists are always looking for a better alternative. 

The natural products always served as a trustable source for new compounds with antiviral properties. Many studies have been carried out since 1995 to isolate bioactive antiviral compounds from plants and functional foods. Accordingly, a large number of plant-derived anti-HSV drugs have been described in several studies. A list of plant species with inhibition studies is summarized in Table 3.

Many herbal compounds have been investigated in the past for their effectiveness against HSV. The purification of new lead compounds from the plants and evaluating their targets and mechanism of action in HSV is also equally important. Many secondary metabolites have been proven to have anti-HSV effects such as lignans, tannins, saponins, terpenes, alkaloids, quinones, and glucosides [155,156,157,158]. In Table 4, we have mentioned the compounds that exhibited viral inhibition with inhibitory activity at the early phase and late phase of replication and HSV viral inhibition with IC_50_ dose.

## 5. Influenza Virus

Pandemics are the mainly remarkable appearances of the influenza virus [160]. Three pandemics happened in the previous century: the H1N1 pandemic (1918), the H2N2 pandemic (1957), and the H3N2 pandemic (1968) [161,162]. Influenza is observed nationally and internationally through a multiparty system of surveillance systems distributed worldwide that eventually feeds into the WHO global influenza program [163,164]. The annual incidence is 3.5 million, with more than 250,000 deaths [165]. Alpha-influenzavirus is the primary cause of all the pandemics [166,167]. Various waves of beta-influenzavirus flu were observed in local settings around the world [168].

Influenza virus belongs to Orthomyxoviridae family (RNA viruses), which includes seven genera (Alpha, Beta, Delta, Gamma, Isavirus, Quaranjavirus, and Thogotovirus) [169,170]. Alpha, Beta, Delta, and Gamma caused mammalian flu. There are 18 various hemagglutinin (HA) subtypes and 11 various neuraminidase (NA) subtypes [171,172]. Subtypes are named by combining the H and N numbers—e.g., A(H1N1), A(H3N2). On the other hand, influenza B viruses are classified into two lineages: B/Yamagata and B/Victoria [173,174]. This genetic pattern imitates the altered nature of the antigenic properties of these viruses, and their following outbreak depends upon various factors [174,175]. Influenza B virus was supposed to have a weaker rate of antigenic progression than A and to cause milder sickness than A in the past [176,177].

**Figure 3:**
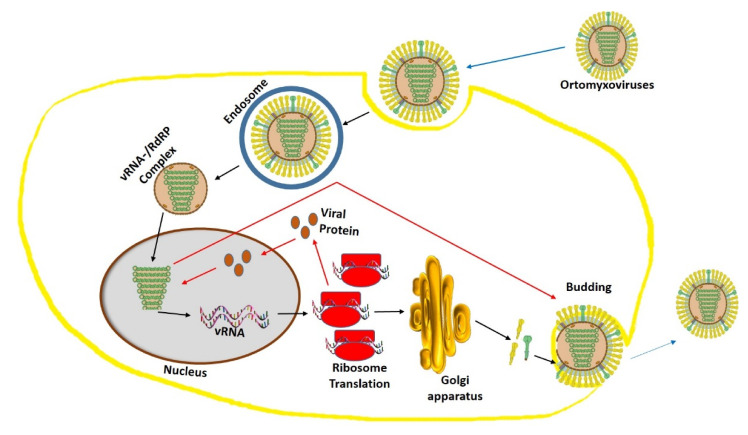
Orthomyxovirus structure and replication mechanism. The Orthomyxovirus structure in this figure has been modified from the source https://viralzone.expasy.org/.

Influenza virus mainly targets the columnar epithelial cells in the respiratory tract [178]. Firstly, the hemagglutinin (HA) present in the receptor binding site of virus attached to galactose bound sialic acid on the surface of the host. This receptor binding is the determining factor for turning part of an organism in a particular direction of infection in response to a virus stimulus. To achieve this receptor binding, the virus undergoes tremendous efforts to bypass host immune responses, mucociliary clearance, and genetic diversification of the host receptor. Then, after the binding, viron enters the host cell by an endocytosis mechanism with the protease cleavage of hemagglutinin. Then, the viron produces a vacuole membrane, which releases the viral RNA and proteins into the cytosol. These proteins and RNA form a complex (vRNA/RdRP), which reaches the nucleus [179,180]. Then, the viral RNA is translated into newly synthesized proteins, which are secreted via the Golgi apparatus to the nucleus to bind viral RNA to form a viral particle. Later, the RNA particle and viral proteins accumulate to form a new viron and buds off from the cell membrane (Figure 3).

In the contingency of a flu pandemic with a new strain, antiviral drugs symbolize the primary line of defense [181,182]. Research on the development of anti-influenza medications was started a long time ago [183,184]. This approach was based on the two mechanisms that induce viral replication in host immune reactions [185,186]. Viral replication has various cellular targets starting from the release of the new viruses from the host cells. Many drugs were scientifically proven to inhibit M2 Ion Channel and Neuraminidase on the virus itself [187,188], while other drugs work on some cell pathways evolving intracellular defense mechanisms [189]. This research on the development of anti-influenza medications also includes identifying traditional medicinal plant extracts and active compounds with anti-influenza activity [190]. These folk drugs were developed as an alternative to synthetic drugs. The exploration of plant-based antivirals against the influenza virus is hopeful, as several plants have been shown to have anti-influenza action. Therefore, the current review paper summarizes the previous findings and efforts of some studies on discovering anti-influenza medications from medicinal plants. A list of plant species with inhibition studies is summarized in Table 5.

Among viral infections, the viruses of the influenza viral infection have the ability to mutate their genome and become resistant to drugs [206]. Thus, the discovery of phytochemicals against the influenza virus is more challenging compared to other viruses. Among the phytochemicals, alkaloids have shown superior activity against flu virus. It is believed that the alkaloids have the ability to kill virus by the induction of interferon of the immune system [207]. Some alkaloids can increase the phagocytosis by macrophages activity, whereas some can inhibit viral protein synthesis [208]. Besides, the inhibition of influenza by lignans [209] and terpenes [210] was well documented. In Table 6, we have mentioned the compounds that exhibited inhibitory activity on viral inhibition with an IC_50_ dose.

## 6. Hepatitis C Virus

Hepatitis C virus (HCV) infection is considered as a significant public health problem. It has infected around 180 million people worldwide [217]. In developed nations, the transmission is thought to be through sharing and the unsafe use of needles among drug users. In the meantime, in the other parts of the world, unsafe blood transfusion and unhealthy injection practices contribute to the development of HCV infection [218]. At present, no vaccine against HCV is available, and the presence of a high diversity of viral isolates will possibly make it very hard to develop a vaccine. Over the last five years, direct-acting antiviral agents (DAAs) have revolutionized the treatment of HCV infection with their specific mechanism of action [219]. DAAs were introduced in 2014, provided effective interferon-free therapy combinations for all HCV genotype, and have very few safety considerations. Serious adverse events are rare, but drug-drug interactions are considered a major issue regarding the choice of DAA regimen, which needs drug-drug interaction assessment before starting therapy [220].

**Figure 4:**
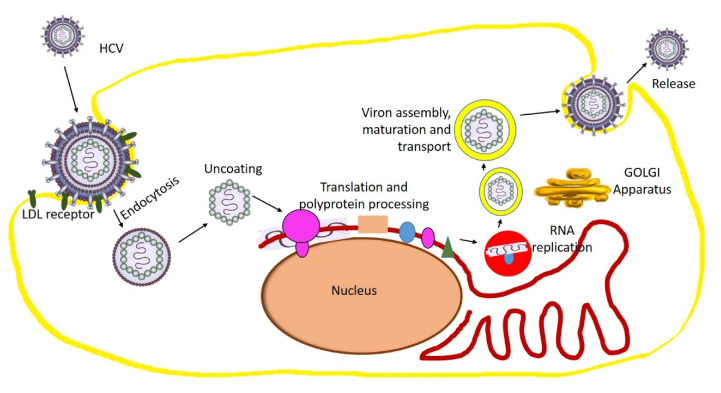
Hepatitis C virus structure and replication mechanism. The Ortomyxovirus structure in this figure has been modified from the source https://www.gettyimages.ae/.

Hepatitis C virus belongs to the Hepacivirus genus of the Flaviviridae family. It is a small enveloped virus with single-stranded genomic RNA with two embedded viral glycoproteins [221]. In the perisinusoidal space (between hepatocyte and a sinusoid), the lipo-viral particle is attached to the basolateral surface of the hepatocyte by virtue of a variety of receptors such as proteoglycans, LDL receptor, CD81, and claudin 1. After the endocytosis, the M2 proteins allow a pH-dependent fusion with the lysosome and the protons to move through the viral envelope, causing the uncoating and release of the viral RNA. Then, the viral replication proteins recruit membranes from the Endoplasmic Reticulum (ER) to form the closely ER-associated “Membranous web”, which is the site of viral replication. Afterward, the viral particles will remain in the nucleus or move to the cytosol, where they are translated into viral proteins via the Golgi apparatus. In addition, the viral proteins sometimes are brought back into the nucleus, where they bind with viral RNA and later form new viral genome particles [222,223]. The new virion buds off from the cell in a phospholipid sphere and is released from the cell (Figure 4).

There are synthetic agents available now against HCV, but they have a lack of specific treatment for HCV therapy. Another concern in these cases is the presence of severe side effects and reported poor response rates. To manage and to get these problems under control for better treatment against HCV, new potential agents to be explored. As we see in the cases of other viruses discussed in the review, there are many promising natural products, which have led to the discovery of potent HCV inhibitors. A list of plant species with inhibition studies is summarized in Table 7.

Developing an anti-HCV drug has become an important priority due to the complexity of the disease. Natural compounds always serve as a lead to create new drugs. There is a substantial increase in the reports on phytochemicals that show anti-HCV properties. Both primary and secondary metabolites have shown promising activities. For instance, alkaloids, flavonoids, polyphenols, coumarins, and peptides have been reported to possess anti-HCV activities [241]. We have identified such molecules and listed them in Table 8.

## 7. Conclusions

Viral infections and pandemic have been recorded as a potential risk for human survival. The lack of proper prophylactic vaccines and drugs for many viruses makes the situation worse in health management. There is a great need for novel antiviral compounds for drug development. This review provides in-depth and insightful information about different species of plants and their families with significant secondary metabolites with evidence-based antiviral properties. Based on the literature, we provided very promising drug candidates that have been investigated through in vitro screening, and cellular targets have been observed. In the current review, we have selected HIV, HSV, HCV and Influenza virus. Looking at the spectrum of plants and isolated compounds, we have seen that there is no significant selectivity among the plants and their compounds in inhibiting DNA or RNA virus. We have found that a similar class of phytochemicals can inhibit both types, but with the ability to inhibit different sites of mechanism. However, these compounds need a lot of further investigation to make them appropriate for clinical use. The pace of new antiviral drugs from natural origin has experienced a substantial upsurge in the last decade. Natural products directly or indirectly support the drug discovery against viruses. Many anti-viral drugs has been discovered from a synthetic source, but originally modeled on a natural product parent structure. Most of the plants we have identified in this review hold other pharmacological benefits, proven long ago, together with their safety profile. This promotes the acceptance of these plants and their phytochemicals for antiviral drug discovery and development programs. Nevertheless, a thorough purification process for identifying new lead compounds and their preclinical and safety testing is a prerequisite. The current COVID-19 pandemic has taught us a more significant lesson: it is difficult to survive in this earth without accepting the probability of more pandemics in the future. Hence, taking the facts in a very comprehensive manner, a cohesive and focused drug discovery approach is warranted.

## Figures and Tables

**Table 1:** Review of the plants that have shown anti-HIV activities with their prospective family, part, type of extract, and inhibition target.

No.	Plant	Family	Part	Extract	Inhibition Target	Ref.
1	*Alchornea laxiflora*	Euphorbiaceae	Root	Methanol	HIV integrase	[31]
2	*Mimusops elengi*	Sapotaceae	Leaf	Ethanol	HIV integrase	[32]
3	*Sceletium tortuosum*	Aizoaceae	Whole plant	Ethanol	HIV integrase	[33]
4	*Hoodia gordonii*	Apocynaceae	Whole plant	Ethanol	HIV integrase	[34]
5	*Panax notoginseng*	Araliaceae	Whole plant	Methanol	HIV integrase	[35]
6	*Arctium lappa*	Asteraceae	Aerial	Methanol	HIV integrase	[35]
7	*Blumea balsamifera*	Asteraceae	Whole plant	Ethanol	HIV integrase	[36]
8	*Chrysanthemum indicum*	Asteraceae	Capitulum	Methanol	HIV integrase	[35]
9	*Chrysanthemum morifolium*	Asteraceae	Capitulum	Ethanol	HIV integrase	[37]
10	*Eclipta prostrate*	Asteraceae	Whole plant	Chloroform	HIV integrase	[27]
11	*Senecio scandens*	Asteraceae	Whole plant	Methanol	HIV integrase	[34]
12	*Boraginaceae Cordia*	Spinescens	Leaf	Methanol, Aqueous	HIV integrase	[38]
13	*Calophyllum inophyllum*	Clusiaceae	Bark	Methanol	HIV integrase	[39]
14	*Dioscorea bulbifera*	Dioscoreaceae	Whole plant	Methanol	HIV integrase	[40]
15	*Albizia procera*	Fabaceae	Whole plant	Methanol	HIV integrase	[35]
16	*Caesalpinia sappan*	Fabaceae	Stem	Methanol	HIV integrase	[35]
17	*Agastache rugosa*	Lamiaceae	Whole plant	Aqueous methanol	HIV integrase	[41]
18	*Salvia miltiorrhiza*	Lamiaceae	Root	Aqueous	HIV integrase	[42]
19	*Lindera aggregate*	Lamiaceae	Stem	Methanol	HIV integrase	[43]
20	*Aglaia lawii*	Meliaceae	Leaf	Methanol	HIV integrase	[44]
21	*Bersama abyssinica*	Melianthaceae	Root	Aqueous	HIV integrase	[45]
22	*Avicennia officinalis*	Acanthaceae	Leaf	Methanol	HIV-reverse transcription	[46]
23	*Justicia gendarussa*	Acanthaceae	Aerial	Ethanol	HIV-reverse transcription	[47]
24	*Rhinacanthus nasutus*	Acanthaceae	Aerial	Hexane	HIV-reverse transcription	[48]
25	*Acorus calamus*	Acoraceae	Rhizome	Hexane	HIV-reverse transcription	[48]
26	*Sambucus nigra*	Adoxaceae	Whole plant	Methanol	HIV-reverse transcription	[49]
27	*Sambucus racemosa*	Adoxaceae	Leaf	Methanol	HIV-reverse transcription	[50]
28	*Aerva lanata*	Amaranthaceae	Root	Hexane	HIV-reverse transcription	[51]
29	*Crinum amabile*	Amaryllidaceae	Bulb	Methanol	HIV-reverse transcription	[52]
30	*Ancistrocladus korupensis*	Ancistrocladaceae	Root	Methanol	HIV-reverse transcription	[53]
31	*Polyalthia suberosa*	Annonaceae	Stem	Methanol	HIV-reverse transcription	[47]
32	*Ridolfia segetum*	Apiaceae	Whole plant	Essential oil	HIV-reverse transcription	[54]
33	*Hemidesmus indicus*	Apocynaceae	Whole plant	Methanol	HIV-reverse transcription	[55]
34	*Tabernaemontana stapfiana*	Apocynaceae	Whole plant	Ethanol	HIV-reverse transcription	[56]
35	*Calendula officinalis*	Asteraceae	Leaf	Dichloromethane	HIV-reverse transcription	[57]
36	*Gamochaeta simplicicaulis*	Asteraceae	Whole plant	Pet ether	HIV-reverse transcription	[58]
37	*Lobostemon trigonus*	Boraginaceae	Whole plant	Aqueous	HIV-reverse transcription	[59]
38	*Brassica rapa*	Brassicaceae	Whole plant	Methanol	HIV-reverse transcription	[60]
39	*Lonicera japonica*	Caprifoliaceae	Flower	Ethanol	HIV-reverse transcription	[61]
40	*Gymnosporia buchananii*	Celastraceae	Whole plant	Methanol	HIV-reverse transcription	[56]
41	*Salacia chinensis*	Celastraceae	Stem	Methanol	HIV-reverse transcription	[48]
42	*Combretum molle*	Combretaceae	Root	Aqueous	HIV-reverse transcription	[62]
43	*Ipomoea aquatic*	Convolvulaceae	Whole plant	80% ethanol	HIV-reverse transcription	[47]
44	*Ipomoea cairica*	Convolvulaceae	Aerial	Water	HIV-reverse transcription	[47]
45	*Ipomoea carnea*	Convolvulaceae	Aerial	Water	HIV-reverse transcription	[47]
46	*Chamaesyce hyssopifolia*	Euphorbiaceae	Whole plant	Methanol	HIV-reverse transcription	[38]
47	*Acalypha Indica*	Euphorbiaceae	Whole plant	Methanol	HIV-reverse transcription	[63]
48	*Euphorbia polyacantha*	Euphorbiaceae	Whole plant	Aqueous	HIV-reverse transcription	[52]
49	*Mallotus philippensis*	Euphorbiaceae	Flower	Methanol	HIV-reverse transcription	[48]
50	*Bauhinia variegata*	Fabaceae	Whole plant	Ethanol	HIV-reverse transcription	[60]
51	*Phaseolus vulgaris*	Fabaceae	Seed	Methanol	HIV-reverse transcription	[64]
52	*Pterocarpus marsupium*	Fabaceae	Whole plant	Aqueous	HIV-reverse transcription	[65]
53	*Tripterospermum lanceolatum*	Gentianaceae	Whole plant	Methanol	HIV-reverse transcription	[66]
54	*Hypericum hircinum*	Hypericaceae	Whole plant	Ethanol	HIV-reverse transcription	[67]
55	*Ajuga decumbens*	Lamiaceae	Whole plant	Methanol	HIV-reverse transcription	[68]
56	*Hyssopus officinalis*	Lamiaceae	Leaf	Methanol	HIV-reverse transcription	[69]
57	*Ocimum kilimandscharicum*	Lamiaceae	Whole plant	Methanol	HIV-reverse transcription	[70]
58	*Ximenia caffra*	Olacaceae	Whole plant	Aqueous	HIV-reverse transcription	[71]
59	*Phyllanthus amarus*	Phyllanthaceae	Whole plant	Aqueous	HIV-reverse transcription	[72]
60	*Scoparia dulcis*	Plantaginaceae	Leaf	Methanol	HIV-reverse transcription	[73]
61	*Canthium coromandelicum*	Rubiaceae	Leaf	Methanol	HIV-reverse transcription	[74]
62	*Alisma plantago-aquatica*	Alismataceae	Rhizome	Aqueous	HIV-protease	[75]
63	*Toxicodendron acuminatum*	Anacardiaceae	Whole	Methanol	HIV-protease	[76]
64	*Xylopia frutescens*	Annonaceae	Bark	Aqueous	HIV-protease	[38]
65	*Ammi visnaga*	Apiaceae	Fruit	Methanol	HIV-protease	[77]
66	*Anethum graveolens*	Apiaceae	Seed	Methanol	HIV-protease	[76]
67	*Angelica grosseserrata*	Apiaceae	Aerial	Aqueous	HIV-protease	[78]
68	*Torilis japonica*	Apiaceae	Seed	Methanol	HIV-protease	[78]
69	*Gymnema sylvestre*	Apocynaceae	Whole plant	Methanol	HIV-protease	[79]
70	*Garcinia buchneri*	Clusiaceae	Steam	Methanol	HIV-protease	[80]
71	*Garcinia kingaensis*	Clusiaceae	Steam	Methanol	HIV-protease	[80]

**Table 2:** Bioactive compounds derived from plants with anti-HIV activities.

No.	Compound	Activity	Dose/IC_50_	Ref.
1	Ellagic acid	Inhibition of HIV integrase	90.23 μM	[30]
2	Gallocatechin	Inhibition of HIV integrase	35.0 µM	[31]
3	Hernandonine	Inhibition of HIV integrase	16.3 μM	[82]
4	Laurolistine	Inhibition of HIV integrase	7.7 μM	[82]
5	7-oxohernangerine	Inhibition of HIV integrase	18.2 μM	[82]
6	Lindechunine A	Inhibition of HIV integrase	21.1 μM	[82]
7	Quercitrin	RT inhibition	60 μM	[83]
8	Gallic acid	Viral infection inhibition	0.36 μg/mL	[84]
9	Erythro-7′-methylcarolignan E	Viral infection inhibition	6.3 μM	[83]
10	Ascalin	RT inhibition	10 μM	[85]
11	Justiprocumins A	RT inhibition	200 μg/mL	[47]
12	Robustaflavone	RT inhibition	65 μM	[86]
13	Hinokiflavone	RT inhibition	65 μM	[86]
14	Agathisflavone	RT inhibition	119 μM	[86]
15	Morelloflavone	RT inhibition	100 μM	[86]
16	Michellamines A	RT inhibition	1 μM	[87]
17	Betulinic acid	RT inhibition	13 μM	[88]
18	Michellamines A2	RT inhibition	29.6 μM	[89]
19	Michellamines A3	RT inhibition	15.2 μM	[89]
20	Michellamines A4	RT inhibition	35.9 μM	[89]
21	Michellamines B	RT inhibition	20.4 μM	[89]
22	Lupeol	RT inhibition	3.8 μM	[55]
23	Lupeol acetate	RT inhibition	6.4 μM	[55]
24	Chlorogenic acid	RT inhibition	4.7 μM	[55]
25	Artemisinin	RT inhibition	100 μM	[90]
26	Luteolin	RT inhibition	12.8 μM	[91]
27	Gossypetin	RT inhibition	2 μg/mL	[92]
28	Xanthohumol	RT inhibition	0.5 μg/mL	[93]
29	Kaempferol 3-rhamnosyl-rutinosid	RT inhibition	0.23 μM	[94]
30	Robustaflavone	RT inhibition	65 μM	[95]
31	Protostanes	RT inhibition	5.8 μg/mL	[96]
32	Morelloflavone	RT inhibition	86 μM	[97]
33	Anolignan A	RT inhibition	156 μg/mL	[95]
34	Cucurbitacins	RT inhibition	28 μM	[98]
35	Oleanolic acid	RT inhibition	2 μg/mL	[99]
36	*p*-cymene	RT inhibition	7.6 μg/mL	[99]
37	Baicalein	RT inhibition	2 μg/mL	[100]

**Table 3:** Review of the plants that show anti-herpes simplex virus activities with their prospective family, part, type of extract, and inhibition target.

No.	Plant	Family	Part	Extract	Mode of Action/Virus	Ref.
1	*Peganum harmala*	Nitrariaceae	Seed	Methanol	Virucidal action/HSV2	[111]
2	*Pistacia vera*	Anacardiaceae	Seed	Methanol	Viral DNA synthesis inhibition/HSV1	[112]
3	*Rhus aromatica*	Anacardiaceae	Root	Aqueous	Inhibit the virus penetration/HSV1	[113]
4	*Quercus brantii*	Cynipidae	Fruit	Chloroform	Inhibit virus entry/HSV1	[114]
5	*Tanacetum parthenium*	Asteraceae	Arial	Aqueous	Virus replication imbibition/HSV1	[115]
6	*Centella asiatica*	Umbelliferae	Aerial	Aqueous	Inhibition of viral replication/HSV2	[116]
7	*Pistacia lentiscus*	Anacardiaceae	Stem	Methanol	Virus absorption imbibition/HSV2	[111]
8	*Mangifera indica*	Anacardiaceae	Leaves	Aqueous	Inhibition of viral replication/HSV2	[116]
9	*Eucalyptus denticulata*	Myrtaceae	Aerial	Acetone	Inhibit virus entry/HSV1	[117]
10	*Aglaia odorata*	Meliaceae	Leaf	Ethanol	Inhibition of viral replication/HSV2	[118]
11	*Euphorbia coopire*	Euphorbiaceae	Flowers	Chloroform/methylene chloride	Inhibition of viral replication/HSV1	[119]
12	*Rhus aromatica*	Anacardiaceae	Bark	Aqueous	Inhibit virus entry/HSV2	[113]
13	*Anacardium occidentale*	Anacardiaceae	Leaf	Aqueous	Inhibition of viral replication/HSV2	[120]
14	*Phoradendron crassifolium*	Loranthaceae	Leaf	Ethanol	Inhibition of viral replication/HSV2	[120]
15	*Morus alba*	Moraceae	Leaf	Aqueous methanol	Inhibition of viral replication/HSV1	[119]
16	*Aloe vera*	Liliaceae	Leaf	Gel	Replication inhibition/HSV1	[121]
17	*Annona muricata*	Annonaceae	Stembark	Petroleum ether	Inhibition of viral replication/HSV2	[122]
18	*Petunia nyctaginiflora*	Solanaceae	Stembark	Petroleum ether	Inhibition of viral replication/HSV2	[122]
19	*Cuphea carthagenensis*	Lythraceae	Ariel	Ethanol	Inhibition of viral replication/HSV1	[123]
20	*Graptopetalum paraguayense*	Crassulaceae	Leaf	Methanol/water	Inhibition of viral replication/HSV1	[124]
21	*Prunus dulcis*	Rosaceae	Almond skin	Methanol/Hcl	Block virus entry	[125]
22	*Equisetum giganteum*	Equisetaceae	Root and stem	Ethanol/water	Inhibition of viral cell attachment and entry/HSV2	[126]
23	*Schinus terebinthifolia*	Anacardiaceae	Bark	Ethanol/water	Inhibition of viral attachment and penetration/HSV1	[127]
24	*Nepeta nuda*	Lamiaceae	Aerial	Aqueous	Inhibition of viral absorption and replication/HSV1	[128]
25	*Cornus canadensis*	Cornaceae	Leaf	Aqueous	Virus absorption inhibition/HSV1	[129]
26	*Strychnos pseudoquina*	Loganiaceae	Stem	Ethyl acetate	Interference with various steps of virus cycle/HSV1	[130]
27	*Tillandsia usneoides*	Bromeliaceae	Fruits	Ethanol	Inhibition of viral replication/HSV1	[123]
28	*Copaifera reticulate*	Fabaceae	Leaf	Ethanol/water	Inhibition of viral cell attachment and entry/HSV2	[126]
29	*Spondias mombin*	Anacardiaceae	Leaf	Methanol	Inhibition of viral cell attachment/HSV1	[131]
30	*Solanum melongena*	Solanaceae	Peel	Ethanol	Reduction of viral protein Expression/HSV1	[132]
31	*Ixeris Sonchifolia*	Compositae	Whole plant	Methanol	Inhibition of viral replication/HSV1	[133]
32	*Eurycoma longifolia*	Simaroubaceae	Stem	Methanol	Inhibition of viral replication/HSV1	[134]
33	*Garcinia mangostana*	Guttiferae	Leaf	Methanol	Inhibition of viral replication/HSV1	[134]
34	*Peganum harmala*	Nitrariaceae	Seed	Methanol	Block virus entry/HSV2	[135]
35	*Erica multiflora*	Ericaceae	Ariel	Methanol	Inhibition of viral replication/HSV1	[136]
36	*Toona sureni*	Meliaceae	Leaf	Methanol	Inhibition of viral replication/HSV1	[134]
37	*Eucalyptus caesia*	Myrtaceae	Aerial	Hydro-distillation	Virucidal activity/HSV1	[137]
38	*Vachellia nilotica*	Fabaceae	Bark	Methanol	Block virus attachment/HSV2	[138]
39	*Stephania cepharantha*	Menispermaceae	Root	Methanol	Virucidal effect/HSV1	[139]
40	*Zygophyllum album*	Zygophyllaceae	Whole plant	Acetone	Virucidal effect/HSV1	[136]
41	*Ficus religiosa*	Moraceae	Bark	Methanol	Virucidal effect/HSV1	[140]
42	*Eucalyptus alba*	Myrtaceae	Fruit	Aqueous	Virucidal effect/HSV1	[134]
43	*Swertia chirata*	Renunculaceae	Leaf	Aqueous	Virucidal effect/HSV1	[141]
44	*Scoparia dulcis*	Plantaginaceae	Leaf	Methanol	Inhibit the viral replication/HSV1	[142]
45	*Pedilanthus tithymaloides*	Euphorbiaceae	Leaves	Methanol	inhibition of viral replication/HSV2	[143]
46	*Melaleuca leucadendron*	Myrtaceae	Fruit	Aqueous	Virucidal effect/HSV1	[134]
47	*Andrographis paniculata*	Acanthaceae	Leaf	Ethanol	Virucidal effect/HSV1	[144]
48	*Artemisia kermanensis*	Asteraceae	Aerial	Hydro-distillation	Virucidal activity/HSV1	[137]
49	*Vigna radiata*	Fabaceae	Spout	Methanol	Virucidal activity/HSV1	[145]
50	*Schleichera oleosa*	Sapindaceae	Fruit	Aqueous	Virucidal activity/HSV1	[134]
51	*Quercus persica*	Fagaceae	Fruit	Hydro alcoholic	Viral attachment inhibition/HSV1	[146]
52	*Pongamia pinnata*	Papillionaceae	Seed	Aqueous	Virucidal activity/HSV1	[147]
53	*Pterocarya stenoptera*	Juylandaceae	Bark	Methanol	Viral attachment and penetration inhibition/HSV2	[148]
54	*Avicennia marina*	Avicenniaceae	Leaf	Methanol	Viral replication inhibition/HSV1	[149]
55	*Nephelium lappaceum*	Sapindaceae	Pericarp	Water/methanol	Virucidal activity/HSV1	[134]
56	*Zataria multiflora*	Labiatae	Aerial	Hydro-distillation	Virucidal activity/HSV1	[137]
57	*Ocimum sanctum*	Lamiaceae	Aerial	Methanol	Viral infection inhibition/HSV1	[150]
58	*Artocarpus lakoocha*	Moraceae	Wood	Methanol	Viral infection inhibition/HSV1	[106]
59	*Scaevola gaudichaudiana*	Asteraceae	Aerial	Dichloromethane	Viral absorption inhibition/HSV1	[151]
60	*Rosmarinus officinalis*	Lamiaceae	Aerial	Hydro-distillation	Virucidal activity/HSV1	[137]
61	*Limonium sinense*	Plumbaginaceae	Root	Ethanol	Virucidal activity/HSV1	[152]
62	*Prunella vulgaris*	Lamiaceae	Fruit spikes	Aqueous	Block HSV-1 binding	[153]
63	*Heterophyllaea pustulata*	Rubiaceae	Fruit	Dried powder	Viral absorption inhibition/HSV1	[154]
64	*Filicium decipiens*	Sapindaceae	Stem bark	Water/methanol	Virucidal activity/HSV1	[134]
65	*Punica granatum*	Punicaceae	Pericarp	Water/methanol	Virucidal activity/HSV1	[134]
66	*Satureja hotensis*	Lamiaceae	Aerial	Hydrodistillation	Virucidal activity/HSV1	[137]

**Table 4:** Bioactive compounds derived from plants with anti-HSV activities.

No.	Compound	Activity	Dose/IC_50_	Ref.
1	4*E*-jatrogrossidentadion	Viral inhibition/HSV 1	2.05 μg/mL	[159]
2	7-galloyl catechin	Viral inhibition/HSV 1	43.2 μg/mL	[119]
3	Gallic acid	Viral inhibition/HSV 1	49.8 μg/mL	[119]
4	Kaempferol 3-*O*-β-(6″-*O*-galloyl)-glucopyranoside	Viral inhibition/HSV 1	124.1 μg/mL	[119]
5	Quercetin 3-*O*-β-(6″-*O*-galloyl)-glucopyranoside	Viral inhibition/HSV 1	175.6 μg/mL	[119]
6	Curcumin	Viral inhibition/HSV 1	49.8 μg/mL	[119]
7	Quercetin	Viral inhibition/HSV 1	78.1 μg/mL	[119]
8	Kaempferol	Viral inhibition/HSV 1	76.1 μg/mL	[119]
9	3,4-Dehydrocycleanine	Viral inhibition/HSV 1	43.2 μg/mL	[139]
10	(−)-Cycleanine	Viral inhibition/HSV 1	26.3 μg/mL	[139]
11	(−)-Norcycleanine	Viral inhibition/HSV 1	18.1 μg/mL	[139]
12	2-Norcepharanoline	Viral inhibition/HSV 1	26.3 μg/mL	[139]
13	Obaberine	Viral inhibition/HSV 1	14.8 μg/mL	[139]
14	Homoaromoline	Viral inhibition/HSV 1	15.1 μg/mL	[139]
15	Aromoline	Viral inhibition/HSV 1	20.4 μg/mL	[139]
16	Isotetrandrine	Viral inhibition/HSV 1	17.4 μg/mL	[139]
17	Berbamine	Viral inhibition/HSV 1	17.4 μg/mL	[139]
18	Thalrugosine	Viral inhibition/HSV 1	16.8 μg/mL	[139]
19	Obamegine	Viral inhibition/HSV 1	23.5 μg/mL	[139]
20	2-Norberbamine	Viral inhibition/HSV 1	16.8 μg/mL	[139]
21	3’,4’-Dihydrostephasubine	Viral inhibition/HSV 1	27.4 μg/mL	[139]
22	Palmatine	Viral inhibition/HSV 1	34.0 μg/mL	[139]
23	Cephakicine	Viral inhibition/HSV 1	44.5 μg/mL	[139]
24	*N*-Methylcrotsparine	Viral inhibition/HSV 1	8.3 μg/mL	[139]
25	Andrographolide	Viral inhibition/HSV 1	8.28 μg/mL	[144]
26	Neoandrographolide	Viral inhibition/HSV 1	7.97 μg/mL	[144]
27	14-Deoxy-11,12-didehydroandrographolide	Viral inhibition/HSV 1	11.1 μg/mL	[144]
28	Oxyresveratrol	Inhibitory activity at the early phase and late phase of replication/HSV1	24 μg/mL	[106]
29	Samarangenin B	Inhibition of viral replication/HSV1	11.4 μg/mL	[152]
30	(−)-epigallocatechin 3-*O*-gallate	Viral inhibition/HSV 1	38.6 μg/mL	[152]
31	Pterocarnin A	Viral attachment inhibition/HSV 1	5.4 μM	[148]
32	Scopadulcic acid B	Viral attachment inhibition/HSV 1	0.012 μM	[142]

**Table 5:** Review of the plants that have shown anti-flu virus activities with their prospective family, part, type of extract, and inhibition target.

No.	Plant	Family	Part	Extract	Inhibition Target	Ref.
1	*Cistus incanus*	Cistaceae	Whole plant	Polyphenol-rich plant extract	MDCK cell-based assay	[191]
2	*Thuja orientalis*	Cupressaceae	Leaves	Methanol	Blockage of attachment to the host cells and inhibition of replication	[192]
3	*Pimpeniella anisum*	Apiaceae	Seeds	Aqueous	Direct effect on replication	[193]
4	*Aloe sinana*	Xanthorrhoeaceae	Root and leaf latex	Methanol	Induced CPE and increased the cell viability of Vero cells	[194]
5	*Punica granatum L.*	Lythraceae	Peel	Ethanol	Inhibit influenza A virus replication	[195]
6	*Geranium thunbergii*	Geranii Herba	Dried aerial part	Ethanol	Neuraminidase (NA) inhibitors	[196]
7	*Mussaenda elmeri*	Rubiaceae	Whole plant	Dichloromethane and methanol in a 1/1 (*v*/*v*) ratio	Inhibition of hemagglutination	[197]
8	*Trigonopleura malayana*	Euphorbiaceae	Leaves	Dichloromethane and methanol in a 1/1 (*v*/*v*) ratio	Inhibition of hemagglutination	[197]
9	*Mussaenda elmeri*	Rubiaceae	Whole plant	Dichloromethane and methanol in a 1/1 (*v*/*v*) ratio	Inhibition of hemagglutination	[197]
10	*Santiria apiculata*	Burseraceae	Whole plant	Dichloromethane and methanol in a 1/1 (*v*/*v*) ratio	Inhibition of hemagglutination	[197]
11	*Anisophyllea disticha*	Anisophylleaceae	Stems	Dichloromethane and methanol in a 1/1 (*v*/*v*) ratio	Inhibition of hemagglutination	[197]
12	*Trivalvaria macrophylla*	Annonaceae	Roots	Dichloromethane and methanol in a 1/1 (*v*/*v*) ratio	Inhibition of hemagglutination	[197]
13	*Baccaurea angulata*	Euphorbiaceae	Stems	Dichloromethane and methanol in a 1/1 (*v*/*v*) ratio	Inhibition of hemagglutination	[197]
14	*Tetracera macrophylla*	Dilleniaceae	Leaves	Dichloromethane and methanol in a 1/1 (*v*/*v*) ratio	Inhibition of hemagglutination	[197]
15	*Calophyllum lanigerum*	Clusiaceae	Whole plant	Dichloromethane and methanol in a 1/1 (*v*/*v*) ratio	Inhibition of hemagglutination	[197]
16	*Calophyllum lanigerum*	Clusiaceae	Stems	Dichloromethane and methanol in a 1/1 (*v*/*v*) ratio	Inhibition of hemagglutination	[197]
17	*Albizia corniculata*	Fabaceae	Stems	Dichloromethane and methanol in a 1/1 (*v*/*v*) ratio	Inhibition of hemagglutination	[197]
18	*Mussaenda elmeri*	Rubiaceae	Whole plant	Dichloromethane and methanol in a 1/1 (*v*/*v*) ratio	Inhibition of hemagglutination	[197]
19	*Polygonum chinense*	Polygonaceae	Whole plant	Methanol	Inhibited viral replication viral neuraminidase	[198]
20	*Bletilla striata*	Orchidaceae	Rhizomes	Ethanol	Viability assay	[199]
21	*Jatropha multifida Linn*	Euphorbiaceae	Stems	70% aqueous ethanol	Virus-infected MDCK cells-based assay	[200]
22	*Dandelion*	Asteraceae	Whole plant	Aqueous	Inhibit polymerase activity and reduce virus nucleoprotein (NP) RNA level.	[201]
23	*Radix Paeoniae Alba*	Paeoniaceae	Roots	Aqueous	Inhibit the replication	[202]
24	*Balanites aegyptiaca,*	Zygophyllaceae	Leaves	Aqueous or 70% methanol	Inhibited the virus-induced hemagglutination of chicken RBCs	[203]
25	*Cordia africana,*	Boraginaceae	Bark	Aqueous or 70% methanol	Inhibited the virus-induced hemagglutination of chicken RBCs	[203]
26	*Aristolochia bracteolata*	Aristolochiaceae	Whole plant	Aqueous or 70% methanol	Inhibited the virus-induced hemagglutination of chicken RBCs	[203]
27	*Boscia senegalensis*	Capparaceae	Leaves	Aqueous or 70% methanol	Inhibited the virus-induced hemagglutination of chicken RBCs	[203]
28	*Leptadenia arborea*	Apocynaceae	Roots	Aqueous or 70% methanol	Inhibited the virus-induced hemagglutination of chicken RBCs	[203]
29	*Punica granatum* *L.*	Lythraceae	Peel	Ethyl alcohol extract	Inhibition of viral adsorption and viral RNA transcription	[204]
30	*Caesalpinia decapetala*	Fabaceae	Leaves	75% aqueous ethanol	Inhibit replication	[205]

**Table 6:** Bioactive compounds derived from plants with anti-flu activities.

No.	Compound	Activity	Dose/IC_50_	Ref.
1	Pentagalloylglucose	Inhibited the virus-induced hemagglutination of chicken RBCs	11.3 µg/mL	[211]
2	Quercetin	Inhibit the entry of the H5N1 virus	7.75 µg/mL	[212]
3	Apigenin	Inhibited viral replication viral neuraminidase	21.54 µM	[213]
4	Baicalein	Inhibited H5N1 viral replication viral neuraminidase	18.79 µM	[213]
5	Biochanin A	Inhibited H5N1 viral replication viral neuraminidase	8.92 µM	[213]
6	Hispidulin	Inhibition against H1N1 neuraminidase	11.18 µM	[214]
7	Nepetin	Inhibition against H1N1 neuraminidase	12.54 µM	[214]
8	Rosmarinic acid methyl ester	Inhibition against H1N1 neuraminidase	15.47 µM	[214]
9	Luteolin	Inhibition against H1N1 neuraminidase	19.83 µM	[214]
10	Homonojirimycin	Inhibition against H1N1 neuraminidase	10.4 µg/mL	[215]
11	Dendrobine	Inhibited early steps in the H1N1 viral replication cycle	3.39 µg/mL	[216]

**Table 7:** Review of the plants that have shown anti-HCV activities with their prospective family, part, type of extract, and inhibition target.

No.	Plant	Family	Part	Extract	Inhibition Target	Ref.
1	*Ajuga bracteosa*	Lamiaceae	Leaves	Methanol	HCV infection Replication	[224]
2	*Ajuga parviflora*	Lamiaceae	Leaves	Methanol	HCV infection Replication	[224]
3	*Berberis lycium*	Lamiaceae	Roots	Methanol	HCV infection Replication	[224]
4	*Toona sureni*	Meliaceae	Leaves	80% Ethanol	HCV infection Replication	[225]
5	*Melicope latifolia*	Rutaceae	Leaves	80% Ethanol	HCV infection Replication	[225]
6	*Melanolepis multiglandulosa*	Euphorbiaceae	Stems	80% Ethanol	HCV infection Replication	[225]
7	*Ficus fistulosa*	Moraceae	Leaves	80% Ethanol	HCV infection Replication	[225]
8	*Phyllanthus amarus*	Phyllanthaceae	Whole plant	Methanol	Inhibition of HCV RNA replication	[226]
9	*Acacia nilotica*	Mimosaceae	Bark	Methanol	Hepatitis C virus (HCV) protease inhibition	[227]
10	*Boswellia carterii*	Burseraceae	Root	Methanol	Hepatitis C virus (HCV) protease inhibition	[227]
11	*Embelia schimperi*	Myrsinaceae	Fruit	Methanol	Hepatitis C virus (HCV) protease inhibition	[227]
12	*Piper cubeba*	Piperaceae	Fruit	Aqueous	Hepatitis C virus (HCV) protease inhibition	[227]
13	*Quercus infectoria*	Fagaceae	Gall	Methanol	Hepatitis C virus (HCV) protease inhibition	[227]
14	*Syzygium aromaticum*	Myrtaceae	Fruit	Aqueous	Hepatitis C virus (HCV) protease inhibition	[227]
15	*Trachyspermum ammi*	Apiaceae	Fruit	Methanol	Hepatitis C virus (HCV) protease inhibition	[227]
16	*Morinda citrifolia*	Rubioideae	Leaves	Methanol	Hepatitis C virus (HCV) protease inhibition	[228]
17	*Silybum marianum*	Asteraceae	Flower	Methanol	Hepatitis C virus (HCV) protease inhibition	[229]
18	*Limonium sinense*	Plumbaginaceae	Flower	Aqueous	HCV infection Replication	[230]
19	*Bupleurum kaoi*	Apiaceae	Root	Methanol	Inhibit HCV entry	[231]
20	*Rhizoma coptidis*	Ranunculaceae	Whole	Methanol	Inhibit HCV entry	[232]
21	*Schisandra sphenanthera*	Schisandraceae	Rhizome	Methanol	Inhibit HCV entry	[232]
22	*Solanum nigrum*	Solanaceae	Seed	Chloroform	NS3 protease inhibition	[233]
23	*Terminalia arjuna*	Combretaceae	Bark	Methanol	NS3 protease inhibition	[226]
24	*Embelia ribes*	Myrsinaceae	Leaf	Aqueous	NS3 protease inhibition	[234]
25	*Aeginetia indica*	Orobanchaceae	Whole	Aqueous	NS5B polymerase inhibition	[235]
26	*Rhodiola kirilowii*	Crassulaceae	Flower	Ethanol	NS3 protease inhibition	[236]
27	*Schisandra sphenanthera*	Schisandraceae	Fruit	Ethanol	Inhibition of HCV entry	[237]
28	*Spatholobus suberectus*	Fabaceae	Leaf	Ethanol	NS3 protease inhibition	[238]
29	*Vitis vinifera*	Vitaceae	Root	Ethanol	NS3 helicase inhibition	[239]
30	*Cinnamomi cortex*	Lauraceae	Bark	Methanol	Inhibition of HCV replication and RNA synthesis	[240]

**Table 8:** Bioactive compounds derived from plants with anti-HCV activities.

No.	Compound	Activity	Dose/IC50	Ref.
1	Embelin	Hepatitis C virus (HCV) protease inhibition	21 µM	[227]
2	Silymarin	NS5B polymerase inhibition	40 µM	[242]
3	5-*O*-Methylembelin	Hepatitis C virus (HCV) protease inhibition	46 µM	[227]
4	Pheophorbide a	Hepatitis C virus (HCV) protease inhibition	0.3 μg/mL	[228]
5	Pentagalloylglucose	Inhibit viral attachment	2.2 µM	[243]
6	Quercetin	inhibitory effect of NS3 catalytic activity	10 µg/mL	[234]
7	Naringenin	Hepatitis C virus (HCV) protease inhibition	200 μM	[244]
8	(+)-Epicatechin	Inhibition of HCV replication	75 μM	[245]
9	(−)-Epicatechin	Inhibition of HCV replication	75 μM	[245]
10	Ladanein	inhibition of the post attachment entry step of HCV	2.5 μM	[246]
11	Luteolin	Inhibition of HCV infection Replication in NS5B polymerase	7.9 μM	[247]
12	Honokiol	Inhibition of HCV infection Replication in NS5B polymerase	4.5 μM	[248]
13	3-Hydroxy caruilignan C	Inhibition of HCV replication	37.5 μM	[249]
14	Gallic acid	Inhibition of viral entry	24.31 μM	[230]
15	Saikosaponin b2	Inhibition of viral entry	16.13 μM	[231]
16	Delphinidin	Inhibition of viral entry	3.7 µM	[250]
17	Amentoflavone	Inhibition of viral entry	42 µM	[251]
18	7,40-Dihydroxyflavanone	Inhibition of viral entry	42 µM	[251]
19	Orobol	Inhibition of viral entry	42 µM	[251]
20	3,3′-Digalloylproprodelphinidin	NS3 protease inhibition	0.77 μM	[236]
21	B2, 3,3′-Digalloylprocyanidin	NS3 protease inhibition	0.91 μM	[236]
22	B2, (−)-Epigallocatechin-3-*O*-gallate, (−)-Epicatechin-	NS3 protease inhibition	8.51 μM	[236]
23	3-*O*-gallate	NS3 protease inhibition	18.55 μM	[236]
24	Schizandronic acid	Inhibition of HCV entry	5.27 μg/mL	[237]
25	Vitisin B	NS3 helicase inhibition	0.006 μM	[239]
26	Procyanidin B1	Inhibition of HCV replication and RNA synthesis	29 μM	[240]
27	Plumbagin	Inhibition of HCV infection Replication in NS5B polymerase	0.57 μM	[252]
28	Caffeine	Inhibition of HCV infection Replication in NS5B polymerase	0.726 mM	[253]
29	Ursolic acid	Inhibition of HCV infection Replication in NS5B polymerase	16 μg/mL	[254]
